# 9-Methoxyellipticine: Antibacterial Bioactive Compound Isolated from *Ochrosia elliptica* Labill. Roots

**DOI:** 10.3390/metabo13050643

**Published:** 2023-05-09

**Authors:** Rana Elshimy, Wael Y. Khawagi, Ibrahim A. Naguib, Sarah I. Bukhari, Riham A. El-Shiekh

**Affiliations:** 1 Department of Microbiology and Immunology, Faculty of Pharmacy, Ahram Canadian University, Giza 12451, Egypt; 2 Department of Microbiology and Immunology, Egyptian Drug Authority, Giza 12511, Egypt; 3 Department of Clinical Pharmacy, College of Pharmacy, Taif University, Taif 21944, Saudi Arabia; 4 Department of Pharmaceutical Chemistry, College of Pharmacy, Taif University, Taif 21944, Saudi Arabia; 5 Department of Pharmaceutics, College of Pharmacy, King Saud University, Riyadh 11451, Saudi Arabia; 6 Department of Pharmacognosy, Faculty of Pharmacy, Cairo University, Kasr el Aini St., Cairo 11562, Egypt

**Keywords:** multidrug resistance, 9-methoxyellipticine, antibacterial activity, nosocomial pathogens, pro-inflammatory factors, carbazole alkaloids

## Abstract

Antibacterial resistance bears a major threat to human health worldwide, causing about 1.2 million deaths per year. It is noteworthy that carbazole derivatives have shown a potential antibacterial activity, for example, 9-methoxyellipticine, which was isolated from *Ochrosia elliptica* Labill. roots (Apocynaceae) in the present study. An in vitro screening of the antibacterial activity of 9-methoxyellipticine was investigated against four multidrug-resistant (MDR) *Klebsiella pneumoniae* and Shiga toxin-producing *Escherichia coli* (STEC O157) as Gram-negative bacteria, in addition to Methicillin-resistant *Staphylococcus aureus* (MRSA) with *Bacillus cereus* as Gram-positive bacteria. The compound had significant antibacterial activity against the two Gram-negative isolates and lower activity against the Gram-positive ones. The synergistic use of 9-methoxyellipticine and antibiotics was successfully effective in reducing the MDR microorganisms. Lung pneumonia and kidney infection mice models were used to investigate the compound’s efficacy in vivo for the first time. Noteworthy reductions in *K. pneumoniae* and STEC shedding and the colonization were observed, with a reduction in pro-inflammatory factors and immunoglobulin levels. Other related lesions such as inflammatory cell infiltration, alveolar interstitial congestion, and edema were noticed to occur, lessened to different limits. The anti-STEC and anti-*K. pneumoniae* activities of 9-methoxyellipticine were revealed, providing a new alternative against MDR nosocomial infections.

## 1. Introduction

Recently, the World Health Organization (WHO) expected that worldwide death triggered by antibacterial-resistant pathogens would extend to 10 million people annually in 2050 [1]. A prospective study of hospital-acquired infections within four tertiary care university hospitals in Egypt within 2 years revealed that 86% and 94.6% of Gram-positive and Gram-negative isolates, respectively, were multidrug resistant (MDR) [2].

Studies indicate that antibiotic resistance is a high priority, markedly in the developing countries, with the exposure to microbial infections at hospitals being a major concern that requires careful study. Recently, the WHO has published a list of “global priority pathogens” (GPP) and indicated the urgent need for the discovery of potent antibacterial agents that can deal with these resistant pathogens [2]. MDR *Klebsiella pneumoniae*, Shiga toxin-producing *Escherichia coli* O157 (STEC O157), Methicillin-resistant *Staphylococcus aureus* (MRSA), and *Bacillus cereus* are considered some of the most serious nosocomial pathogens causing hospital health care problems worldwide. According to the WHO, *K. pneumoniae*, *E. coli*, and *B. cereus* are among the critical pathogens while *S. aureus* is among the high priority pathogens [3].

STEC O157 is one of the major human and animal pathogens worldwide. It can cause several clinical manifestations ranging from stomach cramps and bloody diarrhea to pyelonephritis and hemolytic uremic syndrome (HUS) [2,4]. Additionally, based on the STEC O157 infected humans and animals’ similarity analysis isolates, cross-infection between different hosts was obvious [5]. On the other hand, *K. pneumoniae* is the most common clinical pathogen causing nosocomial infections [6]. Regarding the blood infection pathogens, *K. pneumoniae* is second only to STEC O157, and carbapenem-resistant *K. pneumoniae* that prompts blood infection has a great death rate [7].

The antibacterial research community must incessantly search together to develop new and safer medications to resolve the increasing drug resistance, along with toxicity challenges [8,9,10]. Historically, natural compounds have been a rich source of antibacterial therapies [11,12,13,14]. One class of compounds that should be explored as an anti-bacterial is the alkaloids, such as carbazoles, where the Apocynaceae family presents a high content, particularly in the seeds and latex, and consequently should be explored [15].

Family Apocynaceae (periwinkle family or dogbane family) is one of the largest plant families, with roughly 400 genera and 1500 species. Apocynaceae is a flowering plants family, with trees, shrubs, herbs, or climbers [15].

An example of a plant from the Apocynaceae family is *Ochrosia*. This genus is of about 36 species, which are used as cytotoxic, anti-inflammatory, antioxidant, and anti-malarial agents. *Ochrosia ellpitica* Labill. is a small tropical evergreen shrub native to Oceania in the tribe Vinceae of the subfamily Rauvolfioideae. More than 40 different indole alkaloids have been recognized from the *Ochrosia* species [16,17]. They exhibited several biological activities, such as central nervous system stimulant, cytotoxic, antiseptic, and hypotensive [15,18,19].

In previous work, we were informed that ellipticine, an alkaloid isolated from *Ochrosia ellpitica* Labill. leaves, stopped topoisomerase IV activity, thus killing the multidrug-resistant *E. coli*, and revealed broad-spectrum antimicrobial bioactivities against several bacteria (*Staphylococcus aureus*, *Klebsiella pneumoniae*, *Listeria monocytogenes*, *Pseudomonas aeruginosa*, with *Salmonella typhi*) [20]. Additionally, ellipticines showed the most potent antibacterial activity against *E. coli* and *S. aureus* compared to the studied compounds (MIC < 2 mg/L) with an intact efflux mechanism of action, resulting in their development as effective antibacterial drugs [21]. Among the derivatives of ellipticine tested in this research, ellipticines were discovered with OMe at position 9, which can be beneficial for their antimicrobial action by damaging the bacterial membrane [21]. In addition, the alkaloidal extract of *Ochrosia oppositifolia* was previously examined using disc-diffusion and the calculation of MIC procedures. The results revealed that the highest inhibitory activity against *S. aureus* demonstrated by 10 mg/mL of the investigated leaves and stembark extracts (10.0 ± 2.8 mm and 10.5 ± 2.1 mm, respectively) and the roots against MRSA was 14.0 ± 2.8 mm. Furthermore, the MIC of the mentioned samples against *B. subtilis*, *Salmonella thyphimurium*, and *Serratia marcescens* and the root extract against *Vibrio fluvialis* were 3.75 mg/mL, 0.94 mg/mL, and 0.12 mg/mL, respectively [22].

**Figure 1:**
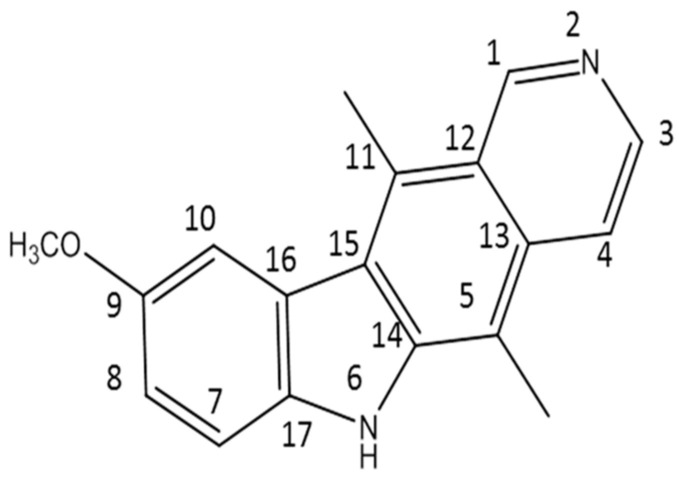
The structure of the isolated compound (9-methoxyellipticine).

In this work, 9-methoxyellipticine (9-methoxy-5,11-dimethyl-6*H*-pyrido [4,3-b]carbazole) (Figure 1) was isolated from *Ochrosia ellpitica* Labill. roots and investigated for its antibacterial potential in vitro towards four of the most common nosocomial agents: *K. pneumoniae*, STEC O157, MRSA, and *B. cereus*. It is worth mentioning that no previous data have been reported about the antibacterial potential of the isolated compound in vivo against *K. pneumoniae* and STEC O157.

## 2. Materials and Methods

### 2.1. Plant Material

*Ochrosia elliptica* Labill. roots were obtained from the Experimental Station of the Faculty of Pharmacy, Cairo University (#28.12.2012 at Pharmacognosy Department, Faculty of Pharmacy, Cairo University).

### 2.2. Extraction and Isolation

The powdered roots (500 g) after air drying were defatted using *n*-hexane (2 × 400 mL) for 48 h by cold maceration in a percolator, then dried, wetted with 25% aqueous ammonia, and left for 2 h. The alkalinized powder was then extracted with dichloromethane (4 × 400 mL). The extract was subjected to acid–base extraction as described by El-Shiekh et al. [15]. Finally, the extract was evaporated to dryness to give an alkaloidal fraction (5 g).

The alkaloidal fraction (5 gm) was subjected to flash column chromatography after application on a PuriFlash column 4100 system (Interchim, Montlucon, France) packed with 30 silica HP. The sample in methanol was applied into the column by the dry load. This was performed using 100% CH_2_Cl_2_ (Fraction 1), followed by increasing the polarity using MeOH in 5–15% increments (Fractions 2–4) and finally 100% pure MeOH (Fraction 5), using 50 mL for each. Fraction 2 (1.5 g) was further chromatographed on a Sephadex LH-20 column (25 × 2.5 cm) using chloroform–methanol (50:50 *v*/*v*) as an eluent to yield a single spot in a subfraction (20–35). This subfraction was collected and dried to give 140 mg of orange powder (Compound 1, R_f_ = 0.37 in chloroform–methanol (95:5 *v/v*)). The yield of the compound was 28 mg/100 g powdered material. The spectral data were compared to the previously published data [15,23]. A Bruker High-Performance Digital FT-NMR Spectrophotometer (Avance III HD), ^1^H-NMR (400 MHz), ^13^C-NMR (100 MHz), Bremen, Germany, using TMS as an internal standard, were used. Chemical shift values were recorded in δ ppm. The NMR Laboratory, Faculty of Pharmacy, Cairo University, hosted the analyses. The data of ^1^H-NMR and ^13^C-NMR were matched with the data published before by El-Shiekh et al. (2017) [16].

### 2.3. Biological Assays

#### 2.3.1. Bacterial Strains

Previously phenotypically and genotypically used bacterial strains MDR STEC O157, MDR *B. cereus*, and MDR *K. pneumoniae* isolated from ICU in a tertiary care center in Cairo [24,25] were used.

#### 2.3.2. Culture Media

Nutrient agar comprising bromocresol purple was used for *B. cereus*, whereas regular nutrient agar was used for the other bacteria. Sabouraud Dextrose Agar (Oxoid Ltd., Thermo Fisher Scientific Inc., Waltham, MA, USA) was employed in the agar diffusion experiments. Furthermore, Mueller–Hinton agar (MHA) medium (Biolab) was used for the disc diffusion assay, though Mueller–Hinton agar containing 0.05% phenol red and supplemented with 10% glucose was used for the minimal inhibition concentration (MIC) tests. TBX chromogenic agar media and MacConkey agar were used for the fecal counting of *E. coli* and *K. pneumoniae*, respectively [26,27].

#### 2.3.3. Screening for Multidrug-Resistant Bacteria

Bacterial cultures were grown at 37 °C overnight on 5% blood agar plates, and then inoculated onto the MHA. The exposures of clinical isolates were performed following the CLSI protocol guidelines for *Enterobacteriaceae* (CLSI, 2018, 2020). Selected MDR bacteria were screened for resistance for more than 2 various classes of antibiotics as mentioned in the disk diffusion method protocol (CLSI, 2020).

#### 2.3.4. In Vitro Antibacterial Assay

##### Agar Disc Diffusion Method

The preliminary screening of the antibacterial activities of 9-methoxyellipticine was first tested for zone of inhibition using the agar disc-diffusion assay according to CLSI guidelines [28]. Standard commercial antibiotic discs, gentamycin (GEN,10 µg) and amikacin (AK,10 µg) for Gram-negative bacteria and vancomycin (VAN,30 µg) and penicillin (PEN,10 µg) for Gram-positive bacteria, were used as a positive control, while a disc impregnated with 0.2% DMSO was used as the negative control.

##### Minimum Inhibitory Concentration (MIC)

The microbroth dilution method was implemented to determine MIC [29]. Two-fold serial dilutions of 9-methoxyellipticine, from 4096 µg/mL to 64 µg/mL (4096, 2048, 1024, 512, 256, 128, and 64 μg/mL), were used. Positive controls (GEN or VAN) were serially diluted from 64 to 0.12 µg/mL. If MIC < 100 µg /mL, the compound was considered significantly active; if MIC < 625 µg /mL, the compound was moderately active, and if MIC ≥ 625 µg /mL, the compound was weakly active [29,30].

#### 2.3.5. In Vivo Study

##### Animals

Eight-week-old male C57BL/6 mice weighing 22 to 25 g were obtained from the Egyptian Drug Authority. In the standard controlled situations, all the animals (n = 10) maintained free access to the foods and water, and were housed in the polyacrylic cage with 24 ± 1 °C temperature, 55 ± 5% humidity, and a 12 h day/night cycle.

##### Bacterial Strains

*K. pneumoniae* was resistant to several antibiotics including AK, ampicillin, flucloxacillin, ciprofloxacin, amoxicillin, meropenem–carbapenem, and levofloxacin and was used in this model to induce lung infection.

*E. coli* STEC O157 was resistant to several antibiotics including AK, cefuroxime, cefazolin, trimethoprim–sulfamethoxazole, chloramphenicol, ciprofloxacin, and tetracycline and was used in this model to induce renal infection.

##### *K. pneumoniae* Mouse Model of Infection

Mice were quarantined for 7 days before infection; the animals were randomized to four groups: sham, 9-methoxyellipticine (5 mg/kg), commercial antibiotic gentamycin (33 mg/kg), and positive control groups. Firstly, to induce *K. pneumoniae* pneumonia infection, all mice were anesthetized by isoflurane inhalation [31]. After holding the mice in an upright position, keeping their heads up, *K. pneumoniae* suspension was trickled into the nasal cavity, with 30 μL (10^8^ CFU/mL) given to all groups except for the sham group [31]. The animals in the sham group were given 30 μL normal saline via the same technique. Subsequently, the animals kept their heads upright for 20 s to ensure the entry of *K. pneumoniae* suspension or normal saline into the lungs by means of gravity [31]. After 30 min of *K. pneumoniae* inoculation, the GEN group (33 mg/kg) and 9-methoxyellipticine group (5 mg/kg/day for three days) were taken intraperitoneally and orally by stomach gavage, respectively. After three days of drug administration, the serum was collected for the biological assessment, in addition to the lungs which were collected for the histopathological examination and scored from (+++) for the maximum severity to (−) for the absence of pathological changes. The mice were sacrificed using cervical dislocation [20].

##### *E. coli* STEC O157 Mouse Model of Infection

The same animal model of infection as described by El-Sheikh et al. (2023) [10] for STEC O1111 was established to investigate the potential of 9-methoxyellipticine against *E. coli* STEC O157.

#### 2.3.6. Ex Vivo Analysis

Sera from the eight groups were stored at −80 °C and the levels of tumor necrosis factor alpha (TNF-α) and immunoglobulin M (IgM) were assessed.

#### 2.3.7. Quantification of Bacterial Shedding of STEC O157

This assay, including the preparation of test samples, initial suspension, and dilution, followed the cited reference [32].

Enumeration: the blue typical colonies (less than 150 CFU) and less than 300 total (typical and non-typical) CFU were counted.

Expression of results: this was determined using the following equation.

$$N=\frac{\Sigma c}{\left(n_{1}+0.1\;n_{2}\right)\;d}$$

Σ*c* is the sum of the characteristic colonies counted on all the dishes retained, *n*_1_ is the number of dishes retained in the first dilution, *n*_2_ is the number of dishes retained in the second dilution, and *d* is the dilution factor corresponding to the first dilution.

## 3. Results and Discussion

9-methoxyellipticine (C_18_H_16_N_2_O, 276.3) is a naturally occurring carbazole alkaloid isolated from *O. elliptica,* which displayed antitumoral, immunosuppressive, and trypanocidal activities [33]. In this study, 9-methoxyellipticine demonstrated favorable activities against STEC, MRSA, MDR *K. pneumoniae*, and MDR *B. cereus* either alone or in combination with a commercial antibiotic when tested with the disc diffusion method.

**Figure 2:**
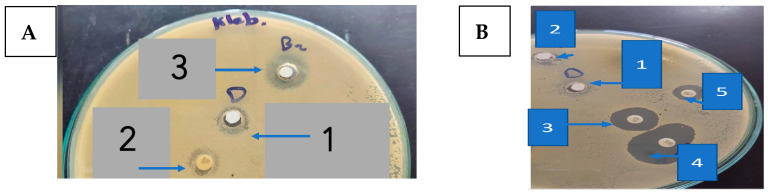
(**A**). The inhibition zone (mm) of 9-methoxyellipticine against *K. pneumoniae* (1: DMSO as negative control; 2: gentamycin as positive control; 3: 9-methoxyellipticine) (x = 3). (**B**). The inhibition zones (mm) of 9-methoxyellipticine against MRSA (1: DMSO as negative control, 2: methoxy ellipticine alone, 3: vancomycin as positive control, 4: vancomycin–9-methoxyellipticine combination, and 5: penicillin–9-methoxyellipticine combination (x = 3).

Regarding STEC O157, GEN showed a strong antibacterial activity with an inhibition zone of 20.0 ± 1.00 mm (Figure 2A and Table 1), while penicillin (PEN) was almost inactive.

One of the highly beneficial methods to guard against the multidrug-resistant bacterial infection is the drug combination [34]. Captivatingly, an excellent activity was detected using 9-methoxyellipticine and both antibiotics—GEN and AK—providing 25.0 ± 0.577 mm and 23.0 ± 1.00 mm as zones of inhibition, correspondingly (Figure 2B and Table 1). Regarding the MDR *K. pneumoniae,* though the strain was resistant to GEN and AK, it was sensitive to 9-methoxyellipticine alone (21 ± 0.577 mm) and in combination with GEN (23.0 ± 1.00 mm) and AK (23.0 ± 1.00 mm) (Figure 2B and Table 1). One possible explanation for that effect might be that 9-methoxyellipticine could result in bacterial cell membrane damage [21], thus improving the efficiency of the antibiotics.

Likewise, 9-methoxyellipticine alone showed an antibacterial inhibition zone of (21 ± 0.577 mm) against MDR *K. pneumoniae* which was GEN resistant (Table 2), while against STEC O157, 9-methoxyellipticine alone showed an inhibition zone of (18 ± 1 mm). On the other hand, 9-methoxyellipticine was ineffective against MRSA and MDR *B. cereus* (3 ± 1.00 mm and 5 ± 0.577 mm, respectively).

For MRSA, the combination of 9-methoxyellipticine and VAN revealed a synergetic antibacterial impact as implied from the growth in the inhibitory zone (from 18 mm to 23 mm) (Table 2). However, the combination of 9-methoxyellipticine with PEN was ineffective in reversing the resistance of both microbes.

The MIC quantities of 9-methoxyellipticine were 512 µg/mL (for *E. coli*) and 256 µg/mL (for MDR K. *pneumoniae*) (Table 2). The compound showed weak activity on Gram-positive bacteria, with an MIC higher than 2048 μg/mL for both MRSA and *B. cereus* (Table 2).

Fortunately, the results are promising for the in vitro bacterial susceptibility test, where against STEC O157 and *Klebsiella pneumoniae* carbapenemase (KPC), 9-methoxyellipticine showed potent bactericidal activity either alone or in combination with commercial antibiotics, such as PEN and GEN, with which 9-methoxyellipticine showed potent synergistic activity.

MIC results for *K. pneumoniae* and STEC O157 were lower than those reported previously by Lu et al. [20] in which MIC ranged from 0.5 and 4 mg/L. This is perhaps due to the difference in chemical structure between 9-methoxyellipticine and ellipticine HCl. This proves that presence of the methoxy moiety is crucial for a potent bactericidal activity.

Upon testing bactericidal activity against MRSA, it was resistant to 9-methoxyellipticine alone but was sensitive when combined with VAN and showed a remarkable synergistic effect by increasing the zone of inhibition from 18.0 ± 1.00 mm to 23.0 ± 1.00 mm. On the other hand, *B. cereus* was totally resistant to 9-methoxyellipticine either alone or when combined with VAN.

According to our results, VAN is recommended to be co-administered with 9-methoxyellipticine during MRSA treatment, highlighting the importance of drug combination when facing MDR strains.

The most common bacterial infections are signified by inflammation caused by lipopolysaccharide (LPS), which is the principle outer membrane component in Gram-negative bacteria and the main role player in the pathophysiological processes of septicemia, toxic shock syndrome, and inflammation [35,36,37].

Nowadays, each infection treatment protocol must contain antibacterial as well as anti-inflammatory agents [20,38]. Previous studies have reported the anti-inflammatory effect of ellipticine against NF-κB [39]. Moreover, the ellipticine that was isolated from the Ochrosia species was labeled as an anti-HIV, anti-inflammatory, and anticancer agent [40,41,42]. The aforementioned studies demonstrated that ellipticine could notably decrease the bacterial loads and tissue destruction in colistin-resistant *E. coli*-infected mice [20], which is in agreement with our report. In our study, 9-methoxyellipticine could effectively lower the levels of TNF-α and IgM, and as a result it could effectively lower lung and kidney damage in *K. pneumoniae* and STEC O157 infected mice, respectively. The positive control groups died prior to the end of the experiment. Analysis experimentation established that all mice were infested with great CFU loads, indicating that mortality rate was triggered by infection.

**Figure 3:**
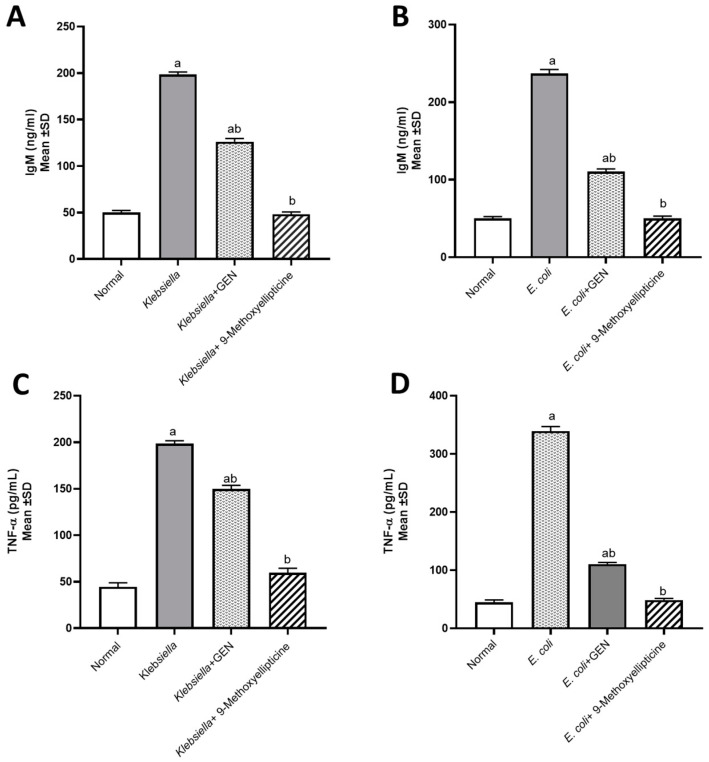
Biochemical assessment of IgM (Immunoglobulin M) in mice infected by MDR *Klebsiella pneumoniae* (**A**) and STEC O157 (Shiga-toxin-producing *Escherichia coli*) (**B**). Biochemical assessment of TNF-α (Tumor necrosis factor alpha) in mice infected by MDR *Klebsiella pneumoniae* (**C**) and STEC O157 (Shiga-toxin-producing *Escherichia coli*) (**D**). ^a^ significant difference from the normal control group at *p* < 0.05. ^b^ Significant difference from the infected group at *p* < 0.05. ^ab^ Significant difference from both normal control and infected groups at *p* < 0.05.

An assessment of TNF-α and IgM levels was performed to prove that *E. coli* and *K. pneumoniae* provoked proinflammatory conditions. However, treatment with 9-methoxyellipticine successfully led to normal levels of TNF-α and IgM being exhibited (Figure 3A–D). In a previous survey, ellipticine notably reduced the levels of pro-inflammatory cytokines and chemokines, resulting in a dramatic reduction in the inflammatory cell infiltration [43]. These findings agreed with our study, as was revealed from biochemical markers and histopathological images and scoring.

Furthermore, natural compounds that function as immunomodulators are needed for improving the immune system, especially in infectious disorders. Ellipticine was reported to have a potent immunostimulant activity [44]. Our report documented the role of alkaloids in immunity, where 9-methoxyellipticine increased the levels of IgM to counter their reduced levels after the infection induced by *K. pneumoniae* and STEC O157 in mice.

**Figure 4:**
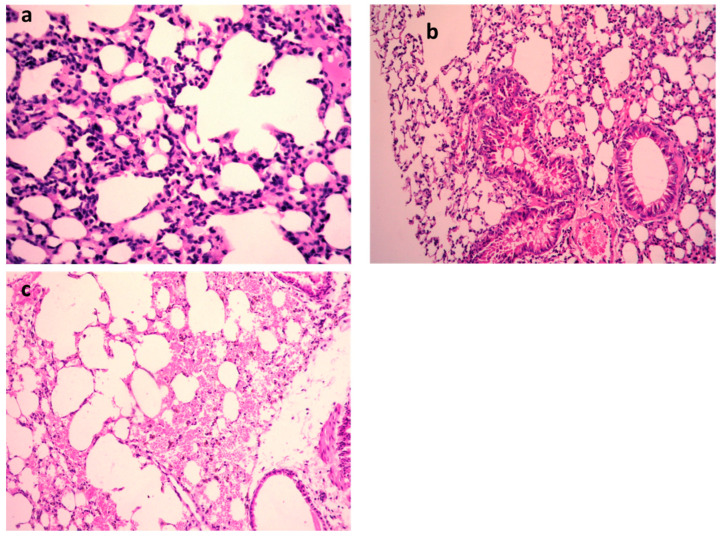
H&E of lungs of the mice infected by MDR *K. pneumoniae*. (**a**) Model group (×400), (**b**) gentamycin (GEN)-treated group (×200), and (**c**) 9-methoxyellipticine-treated group (×400).

*K. pneumoniae* pulmonary lesions (Figure 4a–c) of the untreated positive control exhibited focal hemorrhages in the interstitial tissue and in the alveolar lamina in addition to dilated pulmonary blood vessels. Additionally, inflammatory infiltration composed of the histiocytes and polymorphonuclear sections was seen. In contrast, lungs infected with *K. pneumoniae* subsequently treated by 9-methoxyellipticine revealed the moderate infiltration of inflammatory cells with the emphysema of pulmonary alveoli. Sections of *K. pneumoniae infection* after treatment with GEN demonstrated an emphysema of pulmonary alveoli and a mild inflammatory cell infiltration in the lung tissue (Figure 4a–c). Treatment with 9-methoxyellipticine (Table 3) showed no indication of hemorrhage, no RBCs in alveolar lamina, and no perivascular oedema. More importantly, ellipticine was reported as a suggested treatment for the avoidance of acute death triggered by severe inflammation, with a noteworthy protective activity in *Streptococcus suis serotype 2* strain-infected mice [45].

**Figure 5:**
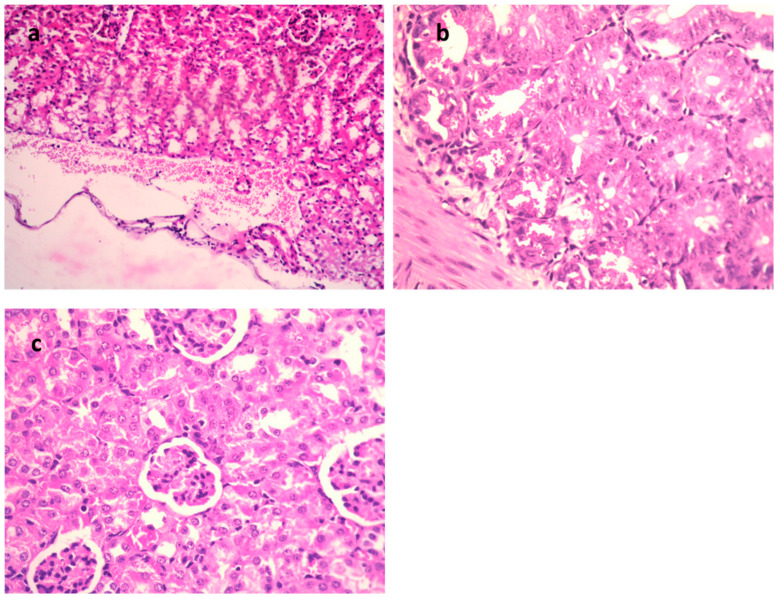
H&E of kidneys of mice infected with MDR *E. coli*. (**a**) Model group (×400), (**b**) gentamycin (GEN)-treated group (×200), and (**c**) 9-methoxyellipticine-treated group (×400).

One day post *K. pneumoniae* inoculation, the infected mice showed indications of quicker respiration, subordinate action, muddled bristle, or coat, and had intensified secretions around the infected eyes (Figure 5a–c and Table 4). Furthermore, these symptoms were faster after 48 h of immunization and the animal started to pass away; all animals died within 7 days in the positive control group, showing that *K. pneumoniae* inoculation causes a critical pulmonary inflammatory response. However, in the 9-methoxyellipticine-treated group, the pulmonary hemorrhage and interalveolar thickness were significantly decreased.

In the STEC-infected animal renal lesions, (Figure 5a–c and Table 4), (untreated group), the manifestation of multi-sized vacuoles in the cytoplasm of tubular epithelium implied necrotic alterations. Additionally, representative of histopathological alteration, coagulation necrosis with karyopyknotic nuclei was also detected. However, the kidney of the animal infected with STEC and treated with 9-methoxyellipticine displayed interstitial inflammatory cell infiltration.

In addition, renal lesions of the animal infected with STEC and treated with GEN showed focal inflammatory cell infiltration in the renal interstitial tissue and deteriorating alterations of the renal tubular epithelium. The histopathological scores (Table 3 and Table 4) demonstrated that mice treated with 9-methoxyellipticine had no renal cast, subcapsular and interstitial hemorrhage, necrobiotic alterations of tubular epithelium, and interstitial inflammatory cell infiltration.

The fecal counts of *E. coli* in animal feces decreased to 5 × 10^1^ after ten days of administration of 9-methoxyellipticine, but those for the GEN-treated animal decreased from 1 × 10^8^ to 2 × 10^3^. Conversely, 9-methoxyellipticine and GEN can decrease the bacterial loads of *K. pneumoniae* from 23 × 10^6^ to 3 × 10^2^ and from 2 × 10^6^ to >220 × 10^2^, respectively (Table 5 and Table 6). In addition to this, the calculation of colonizing *K. pneumoniae* in pulmonary and renal tissues reflected a substantial decline in the bacterial count to 5 × 10^1^ in lungs and to total eradication in kidneys. As is known, MDR STEC infection outbreaks in humans or animals cause a vast risk to public health and safety. Over 700,000 deaths per year globally are due to infection with MDR bacteria [46]. Therefore, finding alternative antibacterial agents with limited side effects has become a must. Lu et al. [20] who tested ellipticine HCl against multidrug-resistant extraintestinal pathogenic *E. coli* reported a MIC nearly half that obtained with 9-methoxy ellipticine (1000 µg /mL for ellipticine HCl and 500 µg /mL for 9- methoxy ellipticine). In addition, the in vivo antibacterial assessment revealed an effective bactericidal activity that was proven by lowering bacterial count to 2 × 10^1^ and 5 × 10^1^ in the lungs and feces, respectively. Therefore, it can be seen that the isolated compound has powerful bactericidal activity. Furthermore, the scoring of histopathological renal and pulmonary lesions revealed a remarkable enhancement in the severity of tissue damage and this indeed proves the ability of 9-methoxy ellipticine to attenuate bacterial pathogenicity.

The extreme inflammatory responses triggered by the bacterial infection are reported as the main causes of the acute death. Collectively, our study demonstrated that 9-methoxyellipticine could substantially decrease the bacterial loads and tissue destruction in MDR STEC O157 and *K. pneumoniae*-infected mice. It also considerably eased the inflammation and pathological injury, with the infiltration of inflammatory cells, alveolar interstitial obstruction, and edema in the pulmonary region and renal tissues of the infected animal. The mechanisms of the antibacterial activity of alkaloids were previously reported [47,48,49].

Finally, 9-methoxyellipticine is a carbazole (nitrogen-containing heterocycle) derivative that showed significant antibacterial activity in our study. As reported, carbazole derivatives exhibit several mechanisms, and can act as antibacterial agents. They act by (1) increasing the membrane permeability through inhibiting specific enzymatic processes and increasing the access of the free radicals, which violates the integrity of the bacterial cells, and (2) interacting with bacterial DNA. Accordingly, various carbazoles are being considered as promising solutions for new antibacterial drugs with two potential actions, thus helping to solve the MDR problems worldwide [50]. Searching for natural compounds could become a promising way to fight against multi-drug resistant bacteria and meet the emergency demand for discovering highly effective, low toxicity, and low environmental impact antibiotics. New antibacterial compounds with novel mechanisms of action and properties that are different from the currently used drugs could be obtained from ellipticine derivatives. In addition, the application of 9-methoxyellipticine as the lead compound is among the most promising approaches for bioactive drug discovery in the future. Finally, a significant clinical impact with the roles of 9-methoxyellipticine and its derivatives in challenging areas should be of great interest in the future as to produce more potent and targeted analogues of 9-methoxyellipticine. Nevertheless, novel strategies based on the field of nanotechnologies for safer drug delivery are recommended.

## 4. Conclusions

This study revealed the promising antibacterial activity of 9-methoxyellipticine using in vitro and in vivo methods, where they successfully showed potential action against MDR *K. pneumoniae* and *E. coli* STEC O157. Moreover, promising results were shown using the combination of our discovered compound with commercial antibiotics against both Gram-positive and Gram-negative bacteria. The scoring of histopathological lesions revealed pronounced improvement after the treatment of the infected groups. Our study extends the bioactivity ability of the isolated compound, with findings that could be extended to achieve pharmacological uses. More 9-methoxyellipticine-based antibiotics are to be investigated in the future to determine the most effective combinations and concentrations. In addition, the structural modifications could amend the pharmacodynamics, pharmacokinetics, and the structure–activity relationship. A synergistic effect with reported treatments should be researched more in order to determine the mechanisms of the anti-bacterial action. Finally, more investigation is required, in particular in vivo, toxicity, and preclinical investigation, with clinical surveys, prior to the classification of the compound as biomedical antibacterial agent.

## Figures and Tables

**Table 1:** Antibacterial activity (Zone of Inhibition) of 9-methoxyellipticine on multidrug-resistant (MDR) Gram-negative bacteria and Gram-positive bacteria.

Sample	Zone of Inhibition in mm (Mean ± SD)			
	STEC O157	MDR *Klebsiella pneumoniae*	*Bacillus cereus*	Methicillin-Resistant *Staphylococcus aureus* (MRSA)
Negative control	0.0 ± 0.0 *	0.0 ± 0.0 *	0.0 ± 0.0 *	9.0 ± 0.0 *
PEN	0.0 ± 0.0 *	6.0 ± 0.0 *	0.0 ± 0.0 *	0.0 ± 0.0 *
GEN	20.0 ± 1.00 ^#^	9.0 ± 1.00 *		
VAN	-	-	9.0 ± 1.00 *	18.0 ± 1.00 ^#^
9-methoxyellipticine	18 ± 1 ^#^	21 ± 0.577 ^#^	5 ± 0.577 *	3 ± 1.00 *
PEN + 9-methoxyellipticine	23.0 ± 0.577 ^#^	22.0 ± 0.577 ^#^	6 ± 0.577 *	11 ± 0.577 *
GEN + 9-methoxyellipticine	25.0 ± 1.00 ^#^	23.0 ± 1.00 ^#^	-	-
VAN + 9-methoxyellipticine	-	-	18.0 ± 1.00 *	23.0 ± 1.00 ^#^

GEN: gentamycin, PEN: penicillin, VAN: vancomycin. * Resistant, ^#^ Sensitive. The experiment was performed in triplicates. SD: standard deviation.

**Table 2:** Minimum inhibitory concentration (MIC) of 9-methoxyellipticine on multidrug-resistant (MDR) Gram-negative and Gram-positive bacteria.

Sample	MIC (µg/mL)			
	Shiga-Toxin-Producing *Escherichia coli* O157 (STEC O157)	MDR *Klebsiella pneumoniae*	Methicillin-Resistant *Staphylococcus aureus* (MRSA)	MDR *Bacillus cereus*
9-methoxyellipticine	512 (1853) *	256 (926.52) *	>2048	>2048
GEN	2 (4.18) *	10 (20.93) *	-	-
VAN	-	-	2	1

GEN: gentamycin, VAN: vancomycin. * The value in uM.

**Table 3:** Scoring lung lesions.

	Normal Control Group	Model Group	9-Methoxyellipticine-Treated Group	Gentamycin (GEN)-Treated Group
Thickened interalveolar septa	−	+++	+	+
Perivascular edema	−	+++	+	−
Hemorrhage	−	+++	−	−
RBCs in alveolar lamina	−	+++	+	−

(+++) severe, (+) moderate, (−) absent.

**Table 4:** Scoring of kidney lesions.

	Normal Control Group	Model Group	9-Methoxyellipticine-Treated Group	Gentamycin (GEN)-Treated Group
Necrobiotic changes in tubular epithelium	−	+++	+	+
Interstitial inflammatory cell infiltration	−	+	−	+
Interstitial hemorrhage	−	+	−	−
Subcapsular hemorrhage	−	+++	−	−
Renal cast	−	−	+	−

(+++) severe, (+) moderate, (−) absent.

**Table 5:** Quantification of bacterial loads of STEC O157 shedding in feces and bacterial colonization in kidney.

	Negative Control	Positive Control STEC O157	STEC O157 and 9-Methoxyellipticine	STEC O157 and GEN	Negative Control
Day zero	2 × 10^4^	3 × 10^6^	4 × 10^4^	4 × 10^6^	36 × 10^1^
Day 4 (before challenge and after treatment with streptomycin)	2 × 10^1^	1 × 10^2^	0	1 × 10^1^	10 × 10^1^
Day 7 (after challenge)	2 × 10^1^	Death	9 × 10^7^	1 × 10^7^	10 × 10^6^
Day10 (after treatment for 3 successive days with GEN i.p., once daily)	2 × 10^1^	Death	10 × 10^3^	2 × 10^3^	7 × 10^8^
Day 17 (after treatment for 7 days with 9-methoxyellipticine orally, once daily)	2 × 10^1^	Death	5 × 10^1^	NA	NA
Kidney	zero	Death	zero	1 × 10^1^	zero

CFU: colony-forming unit, GEN: gentamycin.

**Table 6:** Quantification of bacterial loads of *K. pneumoniae* colonization in lungs (CFU/g Tissue).

	Normal Control	Model Control *K. pneumoniae*	9-Methoxyellipticine-Treated Group	GEN-Treated Group
Lung	*zero*	Death	2 × 10^1^	6 × 10^2^

CFU: colony-forming unit, GEN: gentamycin.

## Data Availability

The data presented in this study are available in article.
